# CytoSorb^®^ Hemadsorption During Microaxial Flow Pump (mAFP) Support in Cardiogenic Shock: A Propensity Score-Matched Cohort Study

**DOI:** 10.3390/biomedicines13102568

**Published:** 2025-10-21

**Authors:** Julian Kreutz, Klevis Mihali, Lukas Harbaum, Georgios Chatzis, Nikolaos Patsalis, Styliani Syntila, Bernhard Schieffer, Birgit Markus

**Affiliations:** Department of Cardiology, Angiology, and Intensive Care Medicine, Philipps-Universität Marburg, Baldingerstraße, 35043 Marburg, Germany

**Keywords:** cardiogenic shock, hemadsorption, microaxial flow pump, inflammation, organ failure

## Abstract

**Background:** Despite advances in temporary mechanical circulatory support (tMCS), patients with cardiogenic shock (CS) who are treated with a microaxial flow pump (mAFP; Impella^®^, Abiomed) still have a high mortality rate. A dysregulated systemic inflammatory response significantly contributes to multiorgan failure in this population. CytoSorb^®^ hemadsorption has emerged as a potential adjunctive therapy for modulating inflammation, but data on its use in CS are limited. **Methods:** This retrospective, single-center study used propensity score matching analysis (1:1 matching; *n* = 15 per group) to compare the outcomes of patients receiving mAFP support with and without concomitant CytoSorb therapy. Baseline data (T0), including comorbidities and clinical status at ICU admission, were collected for all patients. In the CytoSorb group, data were collected at two additional time points: 24 h before the start of CytoSorb therapy (T1), and 24 h after its completion (T2). At these time points, laboratory values and parameters on respiratory, hemodynamic, and organ function were assessed. Corresponding data were also collected for matched patients in the non-CytoSorb group at equivalent time points relative to their matched counterparts. **Results:** In the propensity score-matched cohort, patients treated with CytoSorb exhibited significant improvements between T1 and T2. Specifically, reductions were observed in the vasoactive-inotropic score (*p* = 0.035), procalcitonin levels (*p* = 0.041), peak inspiratory pressure (*p* = 0.036), and positive end-expiratory pressure (*p* = 0.016). Flow rates through the mAFP declined significantly (*p* = 0.014), suggesting stabilization of hemodynamics. These changes were not observed in the non-CytoSorb group, where most parameters remained unchanged or exhibited less pronounced trends. We observed a lower in-hospital mortality rate in the CytoSorb group (33.3% versus 46.7%), though the difference was not significant, potentially due to limited statistical power. **Conclusions:** CytoSorb hemadsorption in mAFP-supported CS was associated with improved hemodynamic stability and reduced inflammatory burden. These findings suggest a potential therapeutic benefit of adjunctive hemadsorption in this high-risk population.

## 1. Introduction

Cardiogenic shock (CS) is a life-threatening condition characterized by inadequate tissue perfusion resulting from severe cardiac impairment [1,2]. Despite significant progress in pharmacological and interventional therapies, CS continues to be associated with high mortality rates, particularly when complicated by systemic inflammation and multiple organ failure [3,4]. In recent years, temporary mechanical circulatory support (tMCS) devices, such as microaxial flow pump (mAFP; Impella^®^, Abiomed), have become an integral part of CS management, providing temporary left ventricular unloading and improving end-organ perfusion [5,6,7]. Hemodynamic stabilization can be provided by tMCS in patients with CS, and the vicious cycle of inflammation, vasoplegia and organ dysfunction may be disrupted by it. In this context, ischaemia–reperfusion injury and shear stress induced by circulatory devices are recognized triggers of systemic inflammation [8]. This inflammatory response can further aggravate hemodynamic instability, impair responsiveness to vasopressors and contribute to progressive multi-organ damage. Targeting this inflammatory component has therefore become a focal point of intensive care research. CytoSorb^®^ is an extracorporeal hemadsorption device designed to reduce systemic hyperinflammation by removing a wide range of circulating mediators [9]. Its porous polymer beads provide a large surface area for the adsorption of hydrophobic molecules in the middle molecular weight range (approximately 5–60 kilodaltons), including cytokines, chemokines, bacterial toxins, myoglobin and bilirubin. This removal process is concentration-dependent, targeting excessively elevated mediators while largely preserving physiological levels. This aims to restore immune balance without inducing immunosuppression. Data from the COSMOS registry indicates that CytoSorb therapy improves hemodynamics, fluid balance and oxygenation in critically ill patients, with observed mortality rates lower than those predicted by baseline severity scores [10]. Integrating it into extracorporeal circuits, such as renal replacement therapy (RRT), or directly into veno-arterial extracorporeal membrane oxygenation (VA-ECMO) systems, offers a potential therapeutic strategy to modulate hyperinflammation in CS. Although case series and observational data have demonstrated promising effects on inflammatory markers and hemodynamics, robust clinical evidence supporting its routine use, especially with concomitant tMCS support, is currently lacking [9,11].

To address this issue, we conducted a retrospective, propensity score-matched cohort study to investigate the impact of CytoSorb therapy in patients with mAFP-supported CS. The study aimed to evaluate changes in inflammatory and hemodynamic parameters, as well as clinical outcomes, to provide real-world insight into the potential role of hemadsorption in this critically ill population.

## 2. Materials and Methods

### 2.1. Study Design and Setting

This is a retrospective, single-center, observational cohort study conducted at the Department of Cardiology, Angiology, and Intensive Care Medicine of the University Hospital of Marburg, Germany. The retrospective analysis was approved by the local Ethics Committee of the Philipps University of Marburg, in compliance with the Declaration of Helsinki (reference: 24-12 RS, approval date: 16 January 2024). Due to the retrospective nature of the study, the Ethics Committee of the Philipps University of Marburg waived the need for obtaining informed consent. Data were collected between 2021 and 2023 from patients treated in intensive care who met the inclusion criteria. All data were derived from routine clinical documentation and handled in compliance with applicable data protection and ethical standards.

### 2.2. Patient Population

Patients were eligible for inclusion in this study if they were 18 years of age or older, fulfilled the diagnostic criteria for CS as defined by the current European Society of Cardiology (ESC) guidelines, and had received mAFP support with Impella CP (Abiomed, Danvers, MA, USA) for a minimum duration of 24 h. Patients were excluded if they had received combined mechanical support with mAFP and VA-ECMO (CardioHelp, Getinge Group, Gothenburg, Sweden), if the mAFP was used solely for elective high-risk percutaneous coronary intervention (PCI) in the absence of CS, or if they had been supported with other mAFP devices such as the Impella 2.5 or Impella 5.5. Furthermore, patients were excluded if relevant clinical and follow-up data were incomplete or missing.

### 2.3. Intervention and Treatment Strategy

Based on the use of concomitant CytoSorb (CytoSorbents Corporation, Princeton, NJ, USA) hemadsorption therapy, patients were assigned to two groups (CytoSorb/non-CytoSorb). The decision to initiate CytoSorb therapy was made at the discretion of the attending physicians, typically in cases of hyperinflammation or progressing organ dysfunction. CytoSorb was either integrated into the extracorporeal RRT circuit or applied in a stand-alone configuration. The treatment lasted at least 24 h, with daily reassessment and replacement of the cartridge every 12 h in accordance with the manufacturer’s recommendations. Anticoagulation was managed with unfractionated heparin, targeting an activated partial thromboplastin time (aPTT) of 50–60 s.

### 2.4. Propensity Score Matching

To minimize selection bias, patients treated with CytoSorb therapy were matched with control patients who did not receive CytoSorb therapy using 1:1 nearest-neighbor propensity score matching without replacement. Propensity scores, which are defined as the predicted probability of receiving CytoSorb therapy given the patient’s baseline characteristics, were estimated using logistic regression. The following baseline characteristics were included in the regression model: dialysis dependency, prior cardiopulmonary resuscitation (CPR), vasoactive-inotropic score (VIS), serum lactate concentration, Horovitz index and Sequential Organ Failure Assessment (SOFA) score at ICU admission. For each CytoSorb patient, the most similar control patient was selected according to the smallest absolute difference in propensity score. Post-matching balance was evaluated using standardized statistical approaches. For continuous variables, independent *t*-tests and Cohen’s d demonstrated excellent comparability: VIS (*p* = 0.967; d = 0.02), lactate (*p* = 0.769; d = 0.11), Horovitz index (*p* = 0.800; d = 0.09) and SOFA score (*p* = 0.442; d = 0.28). Cross-tabulations revealed perfect matching for categorical covariates, with identical distributions for dialysis dependency (14/15 vs. 14/15) and prior CPR (5/15 vs. 5/15).

### 2.5. Data Collection and Outcomes

Clinical and laboratory data were recorded at two predefined time points: Within 24 h prior to the initiation of CytoSorb therapy (T1) and within 24 h following the completion of the initial treatment cycle (T2). These data included laboratory values, respiratory parameters, hemodynamic- and organ function markers. To enable valid comparisons, equivalent data were also collected for matched control patients (non-CytoSorb group) at the same timepoints, based on their matched CytoSorb partner’s individual treatment timeline. Parameters collected included VIS, mAFP flow rates, leukocyte count, procalcitonin levels, albumin concentration, lactate levels, the PaO_2_/FiO_2_ ratio (Horovitz index), and the SOFA score. Clinical outcomes included in-hospital mortality, duration of mechanical ventilation, and RRT dependency at discharge.

### 2.6. Statistical Analysis

Statistical analyses were performed using SPSS Statistics version 29 (IBM Corp., Armonk, NY, USA), R version 4.2.2, and GraphPad Prism version 10 (GraphPad Software, San Diego, CA, USA). Data were tested for normality using the Shapiro–Wilk test. Continuous variables were expressed as the mean ± standard deviation or the median with the interquartile range (IQR), depending on the distribution. Paired or unpaired Student’s *t*-tests were used for normally distributed data, and Mann–Whitney U or Wilcoxon signed-rank tests for non-normally distributed data. Categorical variables were analyzed using the chi-squared test or Fisher’s exact test. Within matched pairs, comparisons were performed using the appropriate paired method. A two-tailed *p*-value of less than 0.05 was considered statistically significant.

## 3. Results

**Figure 1:**
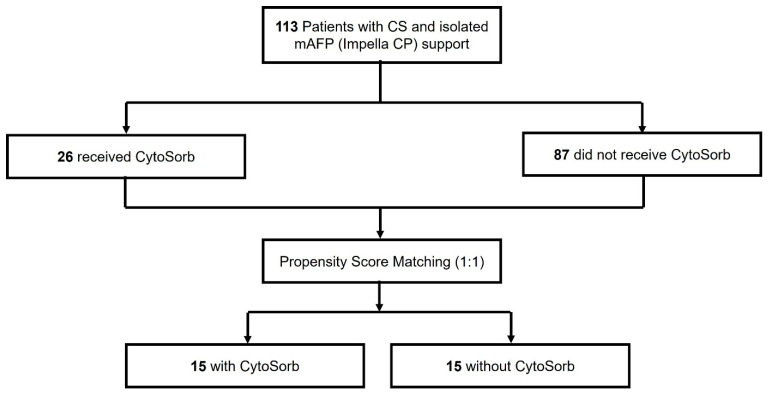
Study Cohort.

During the study period, a total of 144 patients with CS receiving mAFP support were identified at the University Hospital of Marburg. Of these, 31 patients were excluded due to concurrent VA-ECMO therapy. The remaining 113 patients received isolated mAFP support and were included in the subsequent analysis. Among them, 26 patients (23.0%) were treated with CytoSorb hemadsorption, while 87 patients (77.0%) did not receive any hemadsorption therapy. Following 1:1 nearest-neighbor propensity score matching without replacement, 15 matched pairs (*n* = 30) were included in the final comparative analysis (Figure 1).

In the unmatched cohort, no statistically significant differences were observed between the CytoSorb and non-CytoSorb groups concerning baseline demographic characteristics or comorbid conditions (Table 1). For example age, sex distribution, body mass index, prevalence of coronary artery disease, hypertension, diabetes mellitus, and chronic kidney disease were comparable. However, patients treated with CytoSorb presented with substantially greater initial disease severity. This was reflected by significantly higher SOFA scores (mean 12.4 ± 3.1 vs. 7.3 ± 3.7; *p* < 0.001), elevated lactate concentrations (median 3.1 [1.5–6.7] mmol/L vs. 1.5 [1.1–2.1] mmol/L; *p* < 0.001), and markedly increased vasopressor requirements as indicated by VIS (34.4 [21.9–80.2] vs. 8.2 [3.5–17.9]; *p* < 0.001). The degree of pulmonary dysfunction was also more pronounced in the CytoSorb group, as evidenced by a significantly lower Horovitz index (185.3 [146.6–301.3] mm Hg vs. 290.7 [210.3–389.5] mm Hg; *p* < 0.001). The need for multiple organ support further highlighted the clinical instability of this cohort: 96.2% of CytoSorb patients received additional RRT, compared with only 24.1% in the non-CytoSorb group (*p* < 0.001). In addition, the incidence of prior CPR was significantly higher among patients in the CytoSorb group (57.5% vs. 27.6%; *p* = 0.005), underscoring the higher acuity and severity of presentation.

**Figure 2:**
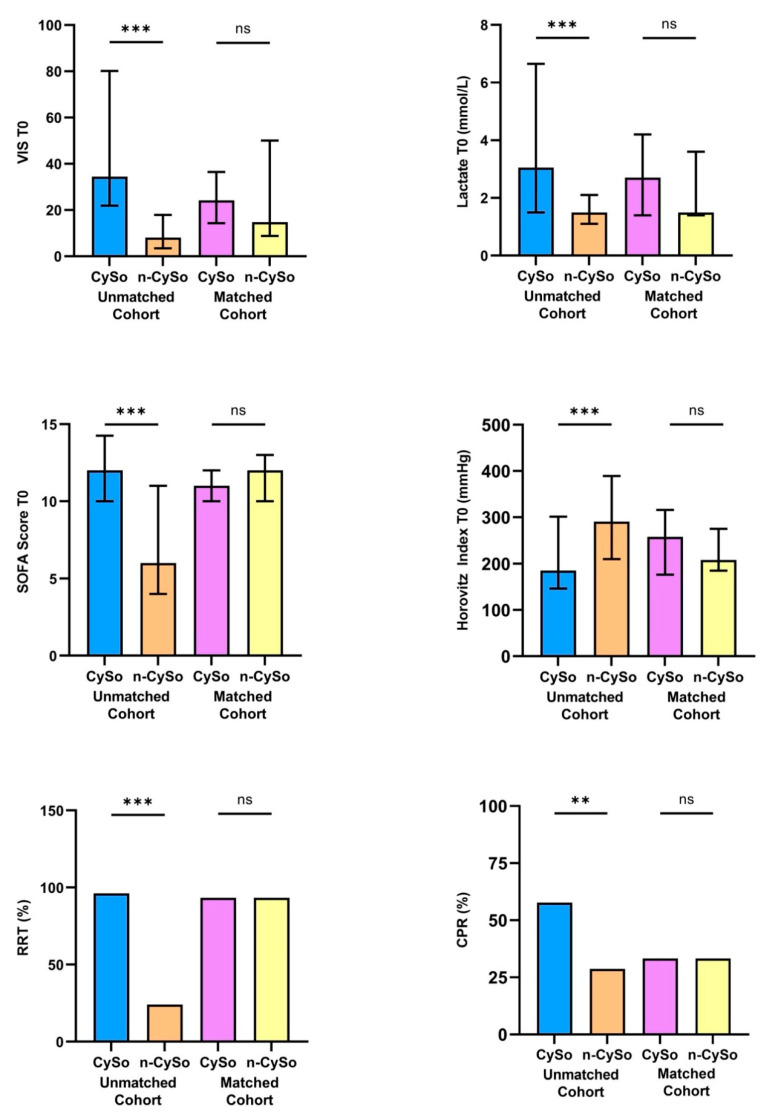
Comparison of key matching variables between patients treated with CytoSorb (CySo) and without CytoSorb (n-CySo) before and after 1:1 matching. ** *p* < 0.01, *** *p* < 0.001, (ns: not significant).

Following 1:1 propensity score matching, the treatment groups were well balanced regarding baseline demographics, comorbidities, and all relevant clinical parameters (Table 1). No statistically significant differences remained in the used key matching variables (Figure 2).

In the matched cohort, clinical outcomes and in-hospital treatment parameters revealed several notable differences between patients treated with CytoSorb and those without CytoSorb (Table 2). Although the difference in overall hospital length of stay did not reach statistical significance, patients in the CytoSorb group exhibited longer duration of hospitalization (28.4 ± 18.2 vs. 19.6 ± 6.3 days; *p* = 0.095). Similarly, the duration of MCS tended to be longer in CytoSorb-treated patients (264.5 ± 17.6 vs. 200.5 ± 13.4 h; *p* = 0.187). The duration of RRT was significantly longer in the CytoSorb^®^ group (median 286.5 [153.3–393.3] hours vs. 161.5 [83.5–277.8] hours; *p* = 0.039). Likewise, patients receiving CytoSorb required significantly longer invasive mechanical ventilation (486.2 ± 308.6 vs. 216.1 ± 163.6 h; *p* = 0.007), despite similar rates of intubation between groups (73.3% vs. 66.7%; *p* = 0.679).

**Figure 3:**
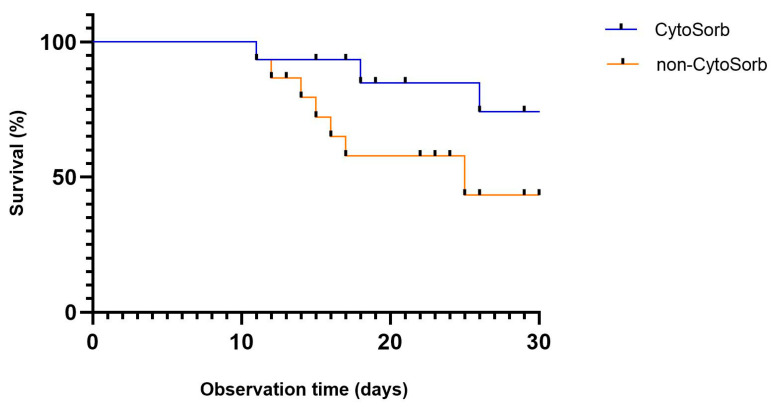
Kaplan–Meier curve showing 30-day survival after propensity score matching in patients with cardiogenic shock treated with or without CytoSorb therapy (*p* = 0.464).

Survival analysis revealed a trend toward improved 30-day survival in the CytoSorb group, as illustrated by the Kaplan–Meier curve (Figure 3). However, the difference in overall mortality between groups did not reach statistical significance (33.3% vs. 46.7%; *p* = 0.464) and was inconclusive due to limited statistical power.

To evaluate the responses to CytoSorb therapy, in both groups laboratory and hemodynamic parameters were assessed in a 24 h period before (T1) and after (T2) treatment (Table 3). The median interval between mAFP implantation and the initiation of CytoSorb therapy was 48 h (IQR 24–96 h). Initiation was determined by the treating physicians according to the patient’s individual clinical trajectory, including signs of hyperinflammation or evolving organ dysfunction.

Inflammatory and laboratory markers revealed a statistically significant decline in procalcitonin (PCT) levels in both groups, with a slightly more pronounced reduction in CytoSorb patients (1.20 [0.5–3.6] to 1.1 [0.4–2.0] ng/mL; *p* = 0.041 vs. 0.9 [0.5–1.2] to 0.9 [0.6–2.2] ng/mL; *p* = 0.006). C-reactive protein levels did not change significantly in the groups. Platelet counts decreased significantly in both cohorts, more markedly in CytoSorb-treated patients (163.0 [146.0–230.0] to 79.0 [55.0–134.0] × 10^9^/L; *p* = 0.004). Metabolic stabilization was observed in both groups; however, to a greater extent in CytoSorb patients. Base excess normalized significantly from −0.9 (−3.1 to 0.8) to 1.8 (−0.7 to 2.7) mmol/L (*p* = 0.005), while lactate levels declined from 1.8 (1.0–2.1) to 1.2 (0.8–1.7) mmol/L (*p* = 0.013). Although arterial pH increased from 7.38 (7.33–7.41) to 7.44 (7.35–7.49), the change did not reach statistical significance (*p* = 0.066). Similar but less pronounced improvements were seen in the non-CytoSorb group.

Respiratory parameters showed a favorable trend during CytoSorb therapy. Both peak inspiratory pressure (pInsp) and positive end-expiratory pressure (PEEP) decreased significantly (pInsp: 20.0 [18.5–25.0] to 18.0 [16.0–22.5] mbar, *p* = 0.036; PEEP: 9.0 [7.5–10.5] to 8.0 [6.5–8.5] mbar, *p* = 0.016), whereas no significant changes occurred in the control group.

**Figure 4:**
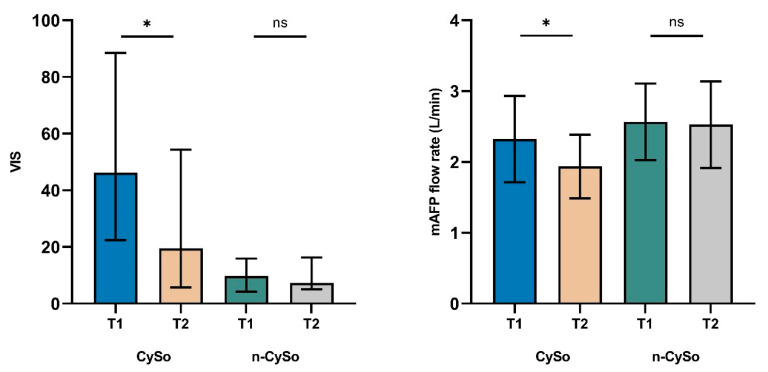
Changes in VIS and mAFP flow rate before (T1) and after (T2) CytoSorb therapy in the matched cohort. Significant reductions were observed in the CytoSorb (CySo) group for both VIS (*p* = 0.035) and mAFP flow rate (*p* = 0.014), with no significant changes in the non-CytoSorb (n-CySo) group. Data are shown as median (IQR) for VIS and mean ± SD for flow rate. * *p* < 0.05. (ns: not significant).

Hemodynamic parameters also improved significantly in the CytoSorb group. The VIS declined from 46.2 (22.4–88.5) to 19.5 (5.8–54.4) (*p* = 0.035), which may reflect a reduced need for pharmacologic circulatory support. In parallel, mAFP flow rates decreased from 2.3 ± 0.6 to 1.9 ± 0.5 L/min (*p* = 0.014), likely reflecting hemodynamic stabilization. No significant changes in VIS or mAFP flow were observed in the control group. In particular, the significant reduction in vasoactive-inotropic requirements and mAFP flow rates, not observed in the non-CytoSorb group, suggests an early hemodynamic response to concomitant CytoSorb therapy (Figure 4).

In contrast, no significant changes in global organ dysfunction scores were observed in either group. SOFA scores remained stable (CytoSorb 12.5 ± 2.0 vs. 12.7 ± 3.2; *p* = 0.788; vs. non-CytoSorb 10.1 ± 3.8 vs. 10.1 ± 4.5; *p* = 0.968), as did oxygenation indices (Horovitz index: CytoSorb 228.3 [180.0–365.7] vs. non-CytoSorb 238.0 [195.1–300.0] mmHg; *p* = 0.460).

## 4. Discussion

In this propensity score matching analysis, we evaluated the clinical impact of concomitant CytoSorb hemadsorption in patients with CS supported by mAFP. While previous observational studies have reported favorable hemodynamic trends in broader tMCS cohorts, our analysis provides a focused insight into the role of extracorporeal cytokine removal during mAFP support [12]. Our findings indicate that CytoSorb therapy is associated with significant improvements in hemodynamic stability, a reduction in systemic inflammation, and enhanced tissue perfusion, along with promising trends toward improved organ function and clinical outcomes. Compared to matched controls, patients treated with CytoSorb demonstrated a statistically significant reduction in VIS after therapy initiation [13,14]. This indicates a more efficient process of weaning off vasopressors and potentially improved myocardial performance under mAFP unloading. CytoSorb has been shown to reduce the levels of cytokines and toxic proteins that cause capillary leakage, vasoplegia and microcirculatory dysfunction [15,16]. This mechanism may also explain the hemodynamic stabilization observed in our mAFP-supported CS cohort. These results are consistent with previous observational data from mixed tMCS cohorts, in which CytoSorb therapy was similarly associated with reduced VIS and improved organ perfusion during mechanical support [12]. However, our analysis focused exclusively on mAFP-supported patients, thereby offering a more targeted perspective on this frequently used MCS modality. Interestingly, the mAFP flow rates were also significantly reduced, suggesting a benefit that may reflect improved myocardial recovery. In our CS patient cohort, CytoSorb treatment was also associated with measurable changes in inflammatory and metabolic parameters. PCT levels declined significantly after initiation of therapy, supporting the hypothesis that extracorporeal hemadsorption can effectively mitigate systemic inflammation [17]. While prior studies have shown clinical benefits in cases of sepsis and acute respiratory distress syndrome, our data on acute myocardial infarction-associated CS support the hypothesis that reducing systemic inflammation in the early phase of CS may facilitate hemodynamic stabilization and prevent secondary organ damage [18,19,20]. However, the underlying etiology of CS may influence the clinical response to hemadsorption. Ischemic shock is primarily caused by ischemia–reperfusion injury and catecholamine-induced inflammation, whereas non-ischemic cardiomyopathies and myocarditis are driven by a variety of immune and structural mechanisms. In inflammatory cardiomyopathies, cytokine activation can be more pronounced, which could enhance the effect of CytoSorb therapy [21,22]. The anti-inflammatory effect may also be relevant for patients with ventricular tachycardia who are at intermediate risk of acute hemodynamic deterioration, beyond overt CS. Recent clinical observations have emphasized the importance of predefined circulatory support strategies for this group of patients [23]. In such cases, the addition of CytoSorb hemadsorption could help stabilize hemodynamics by mitigating inflammation and vasoplegia, potentially modifying the individual’s risk profile.

We also observed significant improvements in pulmonary function, as evidenced by reduced ventilation pressures. These findings may be attributed to decreased capillary leakage and pulmonary congestion in response to reduced systemic inflammation and left ventricular unloading. In addition to modulating inflammatory markers, we also assessed the effects of CytoSorb therapy on hepatic function. Notably, there was a downward trend in hepatic enzymes, including aspartate aminotransferase (AST), alanine aminotransferase (ALT), and lactate dehydrogenase (LDH) during CytoSorb therapy. Although these changes did not reach statistical significance, the observed trends may reflect an attenuation of hepatocellular injury, potentially mediated by improved circulatory stabilization and enhanced microvascular perfusion. Similar findings have been reported in previous studies, where CytoSorb therapy was associated with stabilization or modest improvement of hepatic and cellular injury markers [12,24,25].

Although in-hospital mortality remained considerable in both groups, the CytoSorb group demonstrated a 13.3% absolute reduction in mortality. While this difference did not achieve statistical significance, it suggests a potentially beneficial trend that warrants further investigation. Furthermore, the initiation of CytoSorb was not standardized and varied according to clinical judgment, resulting in heterogeneity in timing and indication. Therefore, patients may have been at different stages of their inflammatory response at T1, which could have influenced the observed effects and limited causal interpretation.

Our results are consistent with those of a recent meta-analysis of septic shock, in which the use of CytoSorb was associated with improved short-term survival and reduced vasopressor requirements [26]. Despite having different causes, both septic and cardiogenic shock involve hyperinflammation and vasoplegia, suggesting a common therapeutic rationale. However, recent analyses have reported decreases in platelets and albumin levels during CytoSorb therapy [27]. Consistently, our cohort also showed a significant reduction in platelet count and serum albumin during treatment. Although a similar trend occurred in the control group, the decline was more pronounced with hemoperfusion. It is thought that device-related platelet loss results from adsorption or activation on the polymer surface, while the reduction in albumin reflects partial protein binding within the cartridge. A post hoc analysis of the Cyto-SOLVE trial revealed that platelet counts decreased significantly during CytoSorb use and that albumin levels dropped in patients with limited substitution, which supports the theory of a device-related effect [27]. Similar findings have been reported in other critically ill patient groups. These effects highlight the importance of regularly monitoring platelet and albumin levels during treatment, and this should be considered when assessing the overall risk–benefit profile of CytoSorb in CS.

The results of this study contribute to the growing body of evidence supporting the use of hemadsorption as a complementary therapy in CS. However, our findings are specific to mAFP-supported patients and should not be generalized to other forms of tMCS without further validation.

## 5. Conclusions

Concomitant use of CytoSorb hemadsorption and mAFP support in CS, appears to improve hemodynamic and inflammatory profiles. Our findings demonstrate significant reductions in vasoactive-inotropic requirements and improvements in pulmonary and organ function within the first 24 h after therapy. While mortality benefits were inconclusive due to limited statistical power, the observed clinical trends are promising and support the integration of hemadsorption into the multimodal management of severe CS. Further prospective randomized studies are needed to confirm these results and establish evidence-based protocols for its use.

## 6. Limitations

This study has several limitations. The retrospective design is inherently subject to selection bias and unmeasured confounding factors. Although propensity score matching was employed to reduce bias, residual confounding cannot be ruled out. Due to the heterogeneous causes of CS, potential differences in treatment response between ischemic, non-ischemic and inflammatory etiologies cannot be ruled out. Further, etiology-stratified studies with biomarker assessment are required to clarify these effects. The small size of the matched cohort (*n* = 15 per group) limits both statistical power and generalizability. The study was not powered to detect differences in mortality or other clinical endpoints. Furthermore, secondary hemodynamic and laboratory findings are exploratory and should be interpreted with caution due to the increased risk of type I and type II errors. CytoSorb therapy was not standardized in terms of timing, duration, or indication. While this reflects real-world variability, it may also introduce heterogeneity in the treatment effect.

## Figures and Tables

**Table 1:** Overview of demographic and baseline characteristics in the overall and matched cohorts. Abbreviations: BMI: body mass index; CAD: coronary artery disease: PTCA: percutaneous transluminal coronary angioplasty, py: pack years, COPD: chronic obstructive pulmonary disease, STEMI: ST-elevation myocardial Infarction, NSTEMI: non-ST-elevation myocardial Infarction. ^1^: *n* (%), ^2^: Mean (SD), ^3^: median (IQR).

		Unmatched Cohort			Matched Cohort		
Variable	All (*n* = 113)	CytoSorb (*n* = 26)	Non- CytoSorb (*n* = 87)	*p*-Value	CytoSorb (*n* = 15)	Non- CytoSorb (*n* = 15)	*p*-Value
**Demographics and comorbidities**							
Age (years) ^2^	68.5 (±12.0)	71.4 (±10.9)	67.6 (±12.2)	0.164	70.8 (±13.2)	70.5 (±10.9)	0.952
Male sex ^1^	88 (77.9)	20 (76.9)	68 (78.1)	0.894	14 (93.3)	12 (80)	0.598
BMI (kg/m^2^) ^2^	27.8 (±4.9)	26.8 (±3.4)	28.1 (±5.2)	0.230	27.1 (±3.5)	27.8 (±3.1)	0.577
CAD with a previous PTCA ^1^	32 (28.3)	9 (34.6)	23 (26.4)	0.419	4 (26.7)	6 (40)	0.446
CAD with a previous bypass surgery ^1^	5 (4.4)	1 (3.8)	4 (4.6)	1.000	1 (6.7)	1 (6.7)	1.000
Arterial hypertension ^1^	75 (66.4)	19 (73.1)	56 (64.4)	0.412	11 (73.3)	10 (66.7)	1.000
Hyperlipidemia ^1^	37 (32.7)	8 (30.8)	29 (33.3)	0.808	5 (33.3)	4 (26.7)	1.000
Diabetes mellitus ^1^	39 (34.5)	10 (38.5)	29 (33.3)	0.631	6 (40)	8 (53.3)	0.472
Insulin-dependent diabetes mellitus ^1^	13 (11.5)	3 (11.5)	10 (11.5)	1.000	1 (6.7)	4 (26.7)	0.330
Nicotine abuse (> 5py) ^1^	41 (36.3)	7 (26.9)	34 (39.1)	0.260	4 (26.7)	6 (40)	0.446
Chronic renal insufficiency KDIGO ≥ stage 3 ^1^	15 (13.3)	3 (11.5)	12 (13.8)	1.000	2 (13.3)	5 (33.3)	0.390
Chronic renal replacement therapy (RRT) ^1^	4 (3.5)	2 (7.7)	2 (2.3)	0.226	2 (13.3)	0 (0)	0.483
COPD ≥ GOLD 2 ^1^	7 (6.2)	3 (11.5)	4 (4.6)	0.198	2 (13.3)	0 (0)	0.483
Asthma ^1^	4 (3.5)	2 (7.7)	2 (2.3)	0.226	2 (13.3)	0 (0)	0.483
Stroke ^1^	9 (8.0)	3 (11.5)	6 (6.9)	0.427	2 (13.3)	2 (13.3)	1.000
Malignant disease ^1^	16 (14.2)	7 (26.9)	9 (10.3)	0.051	4 (26.7)	4 (26.7)	1.000
Peripheral arterial disease ≥ stage 2 ^1^	16 (14.2)	4 (15.4)	12 (13.8)	0.760	1 (6.7)	4 (26.7)	0.330
Heart Failure ≥ NYHA 3 ^1^	16 (14.2)	6 (23.1)	10 (11.5)	0.196	3 (20)	2 (13.3)	1.000
Alcohol Consumption ^1^	5 (4.4)	1 (3.8)	4 (4.6)	1.000	0 (0)	1 (6.7)	1.000
Atrial Fibrillation ^1^	16 (14.2)	3 (11.5)	13 (14.9)	1.000	1 (6.7)	3 (20)	0.598
**Baseline parameters (T0)**							
Vasoactive Score ^3^	10.8 (3.9–31.8)	34.4 (21.9–80.2)	8.2 (3.5–17.9)	<0.001	24.2 (14.3–36.4)	14.8 (8.8–50.0)	0.233
Lactate [mmol/L] ^3^	1.6 (1.2–3.0)	3.1 (1.5–6.7)	1.5 (1.1–2.1)	<0.001	2.7 (1.4–4.2)	1.5 (1.4–3.6)	0.512
SOFA-Score ^2^	8.4 (±4.1)	12.4 (±3.1)	7.3 (±3.7)	<0.001	10.7 (±2.1)	11.3 (±2.2)	0.442
Horovitz Index [mmHg] ^3^	261.6 (189.4–369.8)	185.3 (146.6–301.3)	290.7 (210.3–389.5)	<0.001	257.5 (176.4–316.0)	208.0 (185.0–275.0)	0.775
**Coronary angiography**							
Diagnostic cardiac catheterization during in-hospital stay ^1^	111 (98.2)	26 (100)	85 (97.7)	1.000	15 (100)	15 (100)	1.000
Single-vessel Intervention ^1^	55 (48.7)	10 (38.5)	45 (51.7)	0.237	8 (53.3)	6 (40)	0.472
Multi-vessel Intervention ^1^	39 (34.5)	12 (46.2)	27 (31.0)	0.157	6 (40)	3 (20)	0.427
Coronary intervention ^1^	93 (82.3)	22 (84.6)	71 (81.6)	1.000	14 (93.3)	9 (60)	0.080
STEMI ^1^	49 (43.4)	8 (30.8)	41 (47.1)	0.142	5 (33.3)	3 (20)	0.682
NSTEMI ^1^	40 (35.4)	14 (53.8)	26 (29.9)	0.026	9 (60)	7 (46.7)	0.472

**Table 2:** Data on in-hospital treatment. Abbreviations: ICU: intensive care unit, MCS: mechanical circulatory support, RRT: renal replacement therapy. ^1^: *n* (%), ^2^: Mean (SD), ^3^: median (IQR).

	Matched Cohort		
Variable	CytoSorb (*n* = 15)	Non-CytoSorb (*n* = 15)	*p*-Value
ICU therapy parameters			
Duration of treatment in hospital (days) ^2^	28.4 (±18.2)	19.6 (±6.3)	0.095
Duration of MCS support (hours) ^2^	264.5 (±17.6)	200.5 (±13.4)	0.187
Total time of RRT in ICU (hours) ^3^	286.5 (153.3–393.3)	161.5 (83.5–277.8)	0.039
Invasive ventilation in ICU ^1^	11 (73.3)	10 (66.7)	0.679
Duration of invasive ventilation (hours) ^2^	486.2 (±308.6)	216.1 (±163.6)	0.007
Mortality ^1^	5 (33.3)	7 (46.7)	0.464

**Table 3:** Laboratory and hemodynamic parameters before (T1) and after (T2) CytoSorb therapy in the matched cohort. Abbreviations: ALT: alanine aminotransferase; AST: aspartate aminotransferase; CRP: C-reactive protein; LDH: lactate dehydrogenase; PCT: procalcitonin; PEEP: positive end-expiratory pressure; pInsp: peak inspiratory pressure; SOFA: Sequential Organ Failure Assessment score; VIS: vasoactive-inotropic score. ^2^: Mean (SD), ^3^: median (IQR).

	CytoSorb			Non-CytoSorb		
Variable	T1	T2	*p*-Value	T1	T2	*p*-Value
**Laboratory markers**						
Hemoglobin (g/L) ^3^	90.0 (74.0–99.0)	89.0 (83.0–92.0)	0.460	106.0 (93.0–128.0)	92.0 (81.0–123.0)	0.006
Leukocytes (×10^9^/L) ^3^	14.5 (9.7–18.6)	12.4 (9.1–24.4)	0.826	12.6 (10.3–16.1)	13.1 (11.0–15.2)	0.820
CRP (mg/L) ^3^	15.7 (5.7–21.7)	12.5 (7.3–16.1)	0.820	59.4 (9.2–162.8)	112.6 (11.6–170.4)	0.047
PCT (ng/mL) ^3^	1.2 (0.5–3.6)	1.1 (0.4–2.0)	0.041	0.9 (0.5–1.2)	0.9 (0.6–2.2)	0.006
Platelets (10^3^/L) ^3^	163.0 (146.0–230.0)	79.0 (55.0–134.0)	0.004	172.0 (136.0–235.0)	128.0 (102.0–176.0)	0.001
Total bilirubin (mg/dL) ^3^	0.8 (0.6–1.2)	0.6 (0.5–1.4)	0.975	1.0 (0.7–1.3)	1.0 (0.7–1.6)	0.649
AST (U/L) ^3^	149.0 (49.0–252.0)	72.0 (37.0–252.0)	0.701	110.0 (32.0–286.0)	98.0 (28.0–369.0)	0.211
ALT (U/L) ^3^	56.0 (22.0–173.0)	47.0 (22.0–514.0)	0.233	46.0 (16.0–115.0)	46.0 (14.0–135.0)	0.683
LDH (U/L) ^3^	591.0 (404.0–1771.0)	571.0 (351.0–1286.0)	0.334	528.0 (275.0–796.0)	720.0 (276.0–1496.0)	0.047
Albumin (g/dL) ^3^	26.5 (23.0–30.5)	24.0 (21.0–25.8)	0.028	30.0 (26.0–35.0)	28.0 (25.0–30.0)	0.084
Creatine kinase (U/L) ^3^	421.0 (159.0–1760.0)	250.0 (146.0–1023.0)	0.069	251.0 (149.0–1039.0)	255.0 (128.0–1297.0)	1.000
Myoglobin (ng/mL) ^3^	511.0 (181.0–1597.0)	419.0 (136.0–942.0)	0.061	411.0 (218.0–895.8)	292.5 (178.5–715.3)	0.397
pH ^3^	7.38 (7.33–7.41)	7.44 (7.35–7.49)	0.066	7.40 (7.31–7.46)	7.38 (7.33–7.44)	0.530
Base excess (mmol/L) ^3^	−0.9 (−3.1–0.8)	1.8 (−0.7–2.7)	0.005	−3.7 (−8.2–−0.2)	−4.2 (−5.7–−0.5)	0.842
Lactate (mmol/L) ^3^	1.8 (1.0–2.1)	1.2 (0.8–1.7)	0.013	1.6 (1.0–2.6)	1.1 (0.7–1.8)	0.048
**Respiratory parameters**						
PEEP (mbar) ^3^	9.0 (7.5–10.5)	8.0 (6.5–8.5)	0.016	8.0 (7.0–9.0)	8.0 (7.4–9.0)	1.000
pInsp (mbar) ^3^	20.0 (18.5–25.0)	18.0 (16.0–22.5)	0.036	19.0 (18.0–23.0)	20.0 (17.4–22.5)	0.416
**Hemodynamic and organ function parameters**						
mAFP flow rate (L/min) ^2^	2.3 (±0.6)	1.9 (±0.5)	0.014	2.6 (±0.5)	2.5 (±0.6)	0.833
VIS ^3^	46.2 (22.4–88.5)	19.5 (5.8–54.4)	0.035	9.7 (4.2–15.9)	7.3 (5.0–16.3)	0.152
Horovitz index (mmHg) ^3^	228.3 (180.0–365.7)	238.0 (195.1–300.0)	0.460	256.0 (176.7–452.9)	283.7 (214.0–413.3)	0.910
SOFA-Score ^2^	12.5 (±2.0)	12.7 (±3.2)	0.788	10.1 (±3.8)	10.1 (±4.5)	0.968

## Data Availability

The original contributions presented in this study are included in the article. Further inquiries can be directed to the corresponding author.
